# Supplementary material for: Neurophysiological signatures of cortical micro-architecture
Source: Nat Commun. 2023 Sep 26;14:6000. doi: 10.1038/s41467-023-41689-6 (PMC10522715; doi:10.1038/s41467-023-41689-6)
Supplement: Supplementary file 3 — Description of Additional Supplementary Files [file 41467_2023_41689_MOESM3_ESM.pdf]

Description of Additional Supplementary Files:

Supplementary Dataset 1: provides the full list of hctsa time-series features, and their corresponding PCA loadings and p-values for the PC1 map obtained for the Schaefer100 atlas (Figure 3).

Supplementary Dataset 2: provides the full list of hctsa time-series features and their corresponding PLS loadings for the first latent variable obtained for the Schaefer-100 atlas (Figure 4).

Supplementary Dataset 3: provides the full list of hctsa time-series features, and their corresponding PCA loadings and p-values for the PC1 map obtained for the Schaefer400 atlas (Supplementary Figure 10).

Supplementary Dataset 4: provides the full list of hctsa time-series features and their corresponding PLS loadings for the first latent variable obtained for the Schaefer-400 atlas (Supplementary Figure 11).

Supplementary Dataset 5: provides the full list of hctsa time-series features, their Spearman correlation coefficients with T1w/T2w and their corresponding p-values (Supplementary Figure 7).

Supplementary Dataset 6: provides the full list of hctsa time-series features, their Spearman correlation coefficients with gene expression PC1 and their corresponding pvalues (Supplementary Figure 7).
