# Supplementary material for: Neurophysiological signatures of cortical micro-architecture
Source: Nat Commun. 2023 Sep 26;14:6000. doi: 10.1038/s41467-023-41689-6 (PMC10522715; doi:10.1038/s41467-023-41689-6)
Supplement: Supplementary file 10 — Reporting Summary [file 41467_2023_41689_MOESM10_ESM.pdf]

nature portfolio

Corresponding author(s): Bratislav Misic

Last updated by author(s): Aug 17, 2023

Reporting Summary

Nature Portfolio wishes to improve the reproducibility of the work that we publish. This form provides structure for consistency and transparency in reporting. For further information on Nature Portfolio policies, see our Editorial Policies and the Editorial Policy Checklist.

Statistics

For all statistical analyses, confirm that the following items are present in the figure legend, table legend, main text, or Methods section.

n/a	Confirmed	
☐	☒	The exact sample size (*n*) for each experimental group/condition, given as a discrete number and unit of measurement
☐	☒	A statement on whether measurements were taken from distinct samples or whether the same sample was measured repeatedly
☐	☒	The statistical test(s) used AND whether they are one- or two-sided<br>*Only common tests should be described solely by name; describe more complex techniques in the Methods section.*
☐	☒	A description of all covariates tested
☐	☒	A description of any assumptions or corrections, such as tests of normality and adjustment for multiple comparisons
☐	☒	A full description of the statistical parameters including central tendency (e.g. means) or other basic estimates (e.g. regression coefficient) AND variation (e.g. standard deviation) or associated estimates of uncertainty (e.g. confidence intervals)
☐	☒	For null hypothesis testing, the test statistic (e.g. *F*, *t*, *r*) with confidence intervals, effect sizes, degrees of freedom and *P* value noted<br>*Give P values as exact values whenever suitable.*
☒	☐	For Bayesian analysis, information on the choice of priors and Markov chain Monte Carlo settings
☒	☐	For hierarchical and complex designs, identification of the appropriate level for tests and full reporting of outcomes
☐	☒	Estimates of effect sizes (e.g. Cohen's *d*, Pearson's *r*), indicating how they were calculated

Our web collection on statistics for biologists contains articles on many of the points above.

Software and code

Policy information about availability of computer code

Data collection	MEG data were obtained from the Human Connectome Project (HCP) and were processed using the open software toolbox Brainstorm v220420. (MATLAB)<br>The open source python toolbox neuromaps v0.0.3 was used to compile the micro-architectural feature maps. (Python)<br>Allen Human Brain Atlas (AHBA) data was obtained and processed using the abagen toolbox v0.1.3. (Python)<br>BigBrainWarp toolbox was used to obtain the cortical layer thickness data. (Python)
Data analysis	All code used to process and analyze data can be found at https://github.com/netneurolab/shafiei_megdynamics . All analyses were conducted using Python 3.7.9 , MATLAB R2020a, netneurotools v0.2.3, and other standard Python packages (e.g. Matplotlib, Mayavi, NiBabel, NumPy, Pandas, Scikit-learn, SciPy, Seaborn).<br>Time-series analysis was performed using the highly comparative time-series analysis (hctsa) toolbox v1.07. (Matlab)<br>Spectral parameterization of MEG power was performed using the FOOOF toolbox v1.0.0. (Python)<br>PLS analysis was performed using the pyls package (https://github.com/rmarkello/pyls). (Python)

For manuscripts utilizing custom algorithms or software that are central to the research but not yet described in published literature, software must be made available to editors and reviewers. We strongly encourage code deposition in a community repository (e.g. GitHub). See the Nature Portfolio guidelines for submitting code & software for further information.

Data

Policy information about availability of data

All manuscripts must include a data availability statement. This statement should provide the following information, where applicable:

- Accession codes, unique identifiers, or web links for publicly available datasets
- A description of any restrictions on data availability
- For clinical datasets or third party data, please ensure that the statement adheres to our policy

All data used in the reported analyses are openly available at https://github.com/netneurolab/shafiei_megdynamics. Source data to generate the figures are provided with this paper. The original data used in this study were obtained from the Human Connectome Project (HCP; S900 release) and are publicly available at https://db.humanconnectome.org/. The original HCP data can be accessed following the HCP data use terms. The micro-architectural data is openly available in neuromaps at https://netneurolab.github.io/neuromaps/. The cortical layer thickness data is openly available in BigBrainWarp at https://bigbrainwarp.readthedocs.io/en/latest/. The Allen Human Brain Atlas (AHBA) data is openly available at https://human.brain-map.org. The Schaefer parcellations (i.e., Shcaefer-100 and Schaefer-400 atlases) are openly available at https://github.com/ThomasYeoLab/CBIG/tree/master/stable_projects/brain_parcellation/Schaefer2018_LocalGlobal.

Human research participants

Policy information about studies involving human research participants and Sex and Gender in Research.

Reporting on sex and gender	All the analyses were conducted at the group level. Given the group-level data analysis, sex and/or gender were not considered explicitly in the current report. However, the data used for this study was relatively balanced in sex with 16 female and 17 male participants.
Population characteristics	Healthy young adults (n=33; age range 22-35 years; 16 female/17 male) with no familial relationships from Human Connectome Project (HCP; S900 release)
Recruitment	N/A
Ethics oversight	The WU-Minn HCP Consortium (consortium of US and European institutions led by Washington University and the University of Minnesota) approved the study protocol.

Note that full information on the approval of the study protocol must also be provided in the manuscript.

Field-specific reporting

Please select the one below that is the best fit for your research. If you are not sure, read the appropriate sections before making your selection.

☒ Life sciences ☐ Behavioural & social sciences ☐ Ecological, evolutionary & environmental sciences

For a reference copy of the document with all sections, see nature.com/documents/nr-reporting-summary-flat.pdf

Life sciences study design

All studies must disclose on these points even when the disclosure is negative.

Sample size	No sample size calculations were performed. All analyses were performed at the group level (group-average data), where the data was obtained from participants with no familial relationships from the Human Connectome Project (HCP) database. Specifically, we included all the available participants from the HCP dataset who (1) had MEG scans available, and (2) were not a part of the same family (no familial relationships). This resulted in 33 individuals who were all included in the presented study. In addition, we do not perform any individual-level analysis. All the analyses are conducted at the group-level using group-average data.
Data exclusions	Only MEG data from participants with no familial relationships from the Human Connectome Project (HCP) were included in the analyses.
Replication	Cross-validation analysis was performed for PLS analysis to assess the out-of-sample correlation values. Moreover, all analyses were repeated and the findings were replicated for the Schaefer-100 and Schaefer-400 parcellation resolutions. Finally, the results were replicated using alternative analysis approaches (e.g. spectral parameterization of MEG power, univariate analysis instead of multivariate analysis).
Randomization	No randomization was used given that the present report has no experimental groups.
Blinding	Blinding is not applicable here given that there are no experimental groups in this study.

Reporting for specific materials, systems and methods

We require information from authors about some types of materials, experimental systems and methods used in many studies. Here, indicate whether each material, system or method listed is relevant to your study. If you are not sure if a list item applies to your research, read the appropriate section before selecting a response.

Materials & experimental systems

n/a	Involved in the study
☒	☐ Antibodies
☒	☐ Eukaryotic cell lines
☒	☐ Palaeontology and archaeology
☒	☐ Animals and other organisms
☒	☐ Clinical data
☒	☐ Dual use research of concern

Methods

n/a	Involved in the study
☒	☐ ChIP-seq
☒	☐ Flow cytometry
☒	☐ MRI-based neuroimaging
